# Comparative Analysis of *Sorghum bicolor* Proteome in Response to Drought Stress and following Recovery

**DOI:** 10.1155/2014/395905

**Published:** 2014-10-01

**Authors:** Christoph Jedmowski, Ahmed Ashoub, Tobias Beckhaus, Thomas Berberich, Michael Karas, Wolfgang Brüggemann

**Affiliations:** ^1^Institute of Ecology, Evolution, and Diversity, Johann Wolfgang Goethe-University Frankfurt, Max-Von-Laue Straße 13, 60438 Frankfurt, Germany; ^2^Biodiversity and Climate Research Centre (BiK-F), 60325, Frankfurt am Main, Germany; ^3^Agricultural Genetic Engineering Research Institute (AGERI), ARC, Giza 12619, Egypt; ^4^Institute of Pharmaceutical Chemistry, Johann Wolfgang Goethe-University Frankfurt, 60438 Frankfurt, Germany

## Abstract

The adaptive response of *Sorghum bicolor* landraces from Egypt to drought stress and following recovery was analyzed using two-dimensional difference gel electrophoresis, 2D-DIGE. Physiological measurements and proteome alterations of accession number 11434, drought tolerant, and accession number 11431, drought sensitive, were compared to their relative control values after drought stress and following recovery. Differentially expressed proteins were analysed by Matrix assisted laser desorption ionisation time-of-flight mass spectrometry, MALDI-TOF-MS. Alterations in protein contents related to the energy balance, metabolism (*sensu* Mewes et al. 1997), and chaperons were the most apparent features to elucidate the differences between the drought tolerant and sensitive accessions. Further alterations in the levels of proteins related to transcription and protein synthesis are discussed.

## 1. Introduction

Abiotic stresses like drought, salinity, flooding, temperature extremes, and improper agricultural techniques have a negative impact on final yield of cultivated plants [1]. These stressors apart or combined can result in yield reduction of up to 80% [2]. Water availability is considered a major limitation for plant production [3]. Drought triggers the signal transduction of the phytohormone abscisic acid, ABA, a key hormone involved in stomata closure to reduce transpiration. Drought also suppresses cell growth and photosynthesis efficiency, increases respiration [4], and induces several other genes involved in the response to abiotic stresses [5]. Several drought-inducible genes involved in a broad range of functions have been identified by molecular and genomic analyses in* Arabidopsis*, rice, and other plants [5, 6]. However, the level of a specific mRNA does not always correlate well with the level of proteins. An mRNA produced in abundance may be degraded rapidly or translated inefficiently, resulting in a nonproportional abundance of mRNA and protein. Moreover, out of a given pool of mRNA, only a fraction is recruited further into the polyribosome assembly for translation [7]. Further, many transcripts give rise to more than one protein, through alternative splicing or alternative posttranscriptional modifications. Additionally, many proteins experience posttranslational modifications that profoundly affect their activities. Drought-inducible genes are classified into two groups. The first group includes proteins that function in abiotic stress tolerance such as chaperones, late embryogenesis abundant (LEA) proteins, osmotin, mRNA-binding proteins, key enzymes for osmolyte biosynthesis, water channel proteins, metabolites transporters, detoxification enzymes, and various proteases. Osmotic adjustment including accumulation of sugar alcohols, amino acids, organic acids, and glycine betaine decreases the intracellular osmotic potential. The second group is comprised of regulatory proteins involved in further regulation of signal transduction and stress-responsive gene expression like protein kinases, protein phosphatases, enzymes involved in phospholipid metabolism, and other signaling molecules such as calmodulin-binding protein [8].

*Sorghum bicolor *L. Moench, a C4 grass, ranks the fifth economically important cereal crop worldwide [9]. The availability of the full genome sequence [10] makes sorghum a C4 model plant in addition to the C3 plants* Arabidopsis* and rice to study gene products involved in adaptation to drought stress. In our previous study [11] ten Egyptian genotypes of* Sorghum bicolor* were compared for their drought tolerance using the OJIP test to analyse fast induced chlorophyll fluorescence from photosystem II [12]. In the present study, accession number 11434, drought tolerant, and accession number 11431, drought sensitive, were subjected to proteome analysis using 2D-DIGE system followed by MALDI-TOF-MS. The proteome alteration in response to drought conditions and following watering was compared to their relative controls to figure out difference between drought tolerant and sensitive genotypes.

## 2. Materials and Methods

### 2.1. Plant Materials and Growing Conditions

Accession number 11434, drought tolerant, and accession number 11431, drought sensitive, obtained from the Egyptian National Gene Bank were used in this study. Seeds were germinated in Petri dishes for 48 hours. Three seedlings were planted in 11 × 11 × 11 cm³ plastic pots filled with 700 g soil containing 50% clay, 25% sand, and 25% humus. Ten pots/genotype were used in the experiment. Plants were kept in a greenhouse under 16-hour photoperiod with light intensity of 135 *μ*mol quant m^−2^ s^−1^ at 24°C. Field capacity was adjusted to 70% using an HH2 moisture meter (Delta-T Devices Ltd.). At the stage of 5 extended leaves, further watering was prevented.

### 2.2. Drought and Recovery Treatments

To allow drought adaptive response, the soil was left to dry until the level of 10% field capacity and left further for 7 days without any watering. At the end of drought treatment, the soil water potential was approximately −2 MPa, as measured by a Wescor psychrometer. Following drought treatment, the plants were rewatered till water drain for recovery.

### 2.3. Physiological Measurements

Leaf relative water content, RWC, was measured for plants under full watering regime following drought treatment and 24 hours following recovery according to Smart and Bingham [13]. Fast chlorophyll fluorescence induction curves to calculate Fv/Fm and PI_abs_ as parameters describing the “fitness” of the photosynthetic apparatus [14] were measured between 9 and 10 a.m. following 60 min of dark adaptation on the third leaf of controls (full watered), at the end of the drought treatment, and 24 hours following recovery.

### 2.4. Samples Collection

Following drought treatment, in total 5 third leaves (from one or two out of the three plants per pot) were collected as bulk immediately after chlorophyll fluorescence measurements, frozen under liquid N_2_, and stored at −80°C. 24 hours after recovery, samples of the third leaves were collected from the remaining plants, frozen under liquid N_2_, and stored at −80°C.

### 2.5. Protein Extraction, 2D-DIGE-PAGE, and MALDI-TOF-MS

Protein extraction was carried out using 200 mg tissue following the established 10% TCA-acetone extraction protocol [15] with the modification of replacing the 0.07% 2-mercaptoethanol with 10 mM DTT in the 10% TCA-acetone and absolute acetone. Proteins were resuspended in 1 mL of resuspension solution (7 M urea and 2 M thiourea). Resuspension was achieved with 3 times sonication on ice, 5 seconds each at 35% power output using a Sonopuls mini 20 sonicator, and a MS 1.5 probe (Bandelin). Samples were clarified by two subsequent centrifugations at 13000 rpm for 15 minutes at 20°C. Protein concentration was measured [16] and adjusted to 2 mg mL^−1^ using resuspension solution. An aliquot of 50 *μ*g protein samples was labeled with Cy-2, Cy-3, or Cy-5 for control, drought treated, and recovery treated samples, respectively, using Cy-Dye DIGE Fluor Minimal Dye Labeling Kit (GE Healthcare) following the manufacturer's recommendation. Following labeling, samples were mixed together and 200 *μ*g of unlabeled protein from drought and recovery plant extracts were added to allow sufficient proteins for MALDI-TOF-MS reaction. Samples were adjusted to 360 *μ*L with resuspension solution and 90 *μ*L 5X loading buffer, containing 20% (w/v) CHAPS, 200 mM DTT, 10% (v/v) IPG buffer pH 3–10, and 0.01% (w/v) bromophenol blue, was added. Samples were used to rehydrate 24 cm pH 3–10 nonlinear IEF strips (GE-Healthcare) overnight prior to IEF using the Multiphor II Electrophoresis System (GE-Healthcare) at 70 kV*·*h. Following IEF and prior to separation on a 20 cm length 10% SDS-PAGE [17] casted into low fluorescence glass plates, strips were equilibrated for 15 minutes in 15 mL of SDS equilibration solution (6 M urea, 75 mM Tris-HCl pH 8.8, 29.3% (v/v) glycerol, 2% (w/v) SDS, and 0.002% (w/v) bromophenol blue) containing 1% DTT, followed by additional 15 minutes in SDS equilibration solution containing 2.5% (w/v) iodoacetamide. Following electrophoresis, gels were scanned using a Typhoon 9410 scanner (GE healthcare). Images were analyzed using DeCyder 2-D Differential Analysis Software (GE healthcare). After analysis, gels were stained overnight using colloidal blue stain (Invitrogen). MALDI-TOF-MS was carried out on protein spots excised from polyacrylamide gels as described in Ashoub et al. [18] and the included references. Proteins were identified using Mascot (Matrix Science; peptide mass tolerance: 60 ppm; setting of maximum missed cleavages at 1) using the NCBInr database. The criterion for identifying a protein as significant up- or downregulated was a 1.5-fold threshold change in relative fluorescence signal intensity by pairwise comparison of the dye signals from the three coelectrophoresed samples [19, 20]. Protein MALDI-TOF-MS results with a score above the identification threshold 84 (*P* < 0.05) were considered significant for accuracy of identification.

## 3. Results and Discussion

In this study, a comparison is made between two* Sorghum* genotypes which differ in their response to drought based on the physiological differences of their photosynthetic apparatus. A soil water potential of approximately −2 MPa was applied to ensure that plants perceived severe drought under greenhouse conditions. The 2D-DIGE approach was performed to analyze the changes in the proteome after drought stress and following recovery. The study used the MALDI-TOF-MS analysis to identify differentially expressed proteins.

### 3.1. Physiology

**Figure 1:**
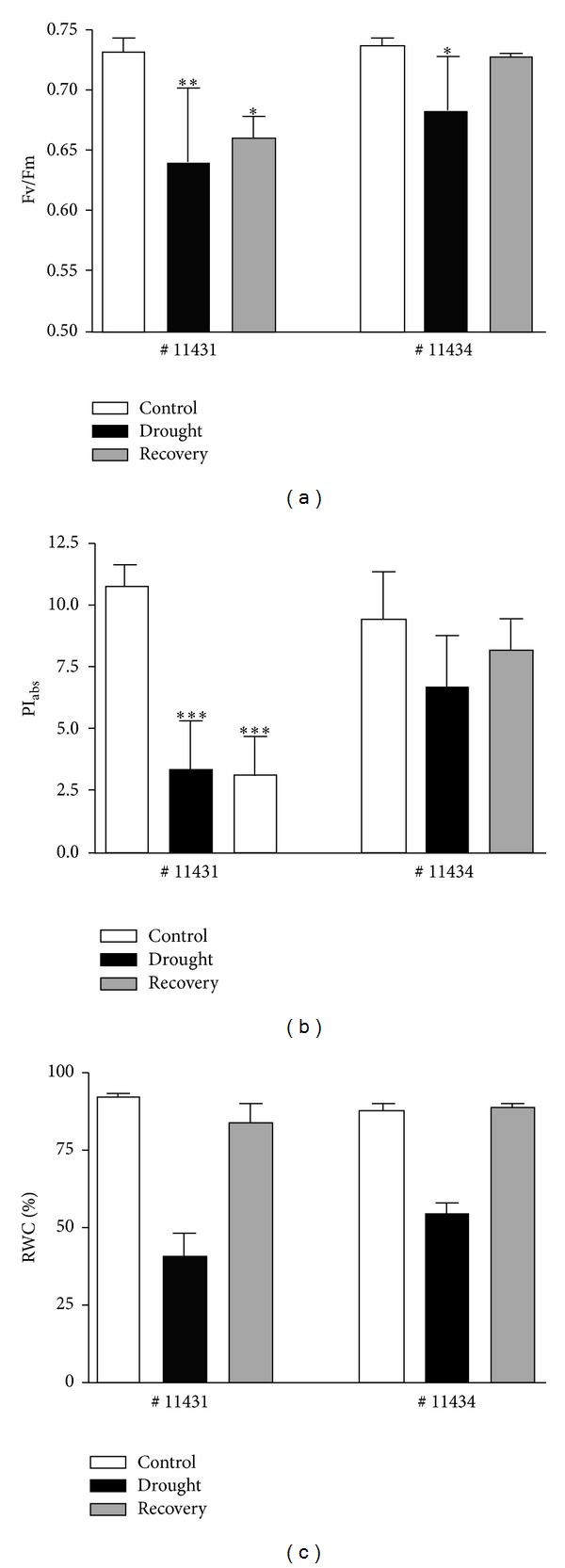
Fv/Fm values (a), PI_abs_ (b), and RWC (c) of # 11434, drought tolerant, and # 11431, drought sensitive, genotypes. Standard deviation was calculated using 5 independent measurements. One-way ANOVA test was used to assess the significance of differences at *P* < 0.001 (∗∗∗), *P* < 0.01 (∗∗), or *P* < 0.05 (∗) between treatments and their respective controls.

The Fv/Fm parameter representing the maximum quantum efficiency of photosystem II [21] and the performance index, PI_abs_, as an indicator of the overall internal strength of the sample to resist external stresses [22] were used as parameters to demonstrate the physiological status of the* Sorghum* genotypes # 11434 (drought tolerant) and # 11431 (drought sensitive) following drought treatment and recovery. The data provided in Figure 1 represent the means of 5 measurements per accession. One-way ANOVA was used to assess the significance of the differences among treatments at each experimental stage, and Dunnett test at *P* < 0.05 separated the means. Figures 1(a) and 1(b) show the data of Fv/Fm and PI_abs_ for both genotypes, respectively. Both genotypes showed a reduction in Fv/Fm and the PI_abs_ values at the end of drought treatment in comparison to their control values, with a smaller effect in # 11434 than in # 11431. After 24 hours of recovery, both Fv/Fm and PI_abs_ values indicated recovery of # 11434 to the relative values of control, while the chl fluorescence parameters in # 11431 had not reached the level of the control values again. RWC was used as a parameter to directly assess the plant water status. RWC was reduced to about 40% (#11431) and 50% (# 11434) following drought treatment, respectively. After 24 hours of rewatering, the initial water content was reached again in both genotypes (Figure 1(c)).

### 3.2. Proteomics

**Figure 2:**
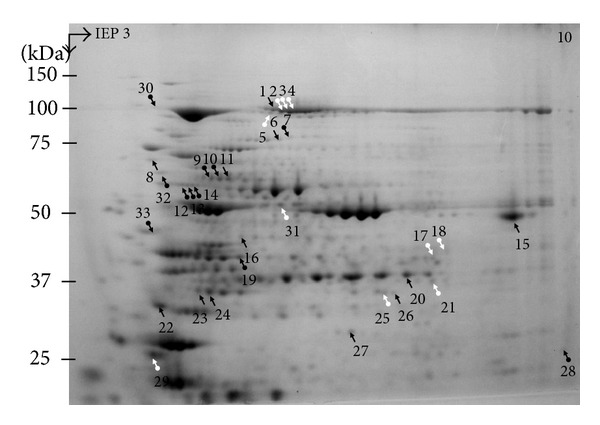
Differentially expressed proteins on 10% SDS-PAGE following separation on 24 cm nonlinear strips pH 3–10, scanning, and staining with Colloidal Coomassie Brilliant Blue. Black arrows represent proteins expressed in both genotypes. Black arrows with round bottom represent expressed proteins in # 11434, drought tolerant genotype, and white arrows with round bottom represent expressed proteins in # 11431, drought sensitive, genotype.

In the proteome analysis experiments, we used the Cy-2, Cy-3, and Cy-5 dyes for labeling protein extracts of control, drought, and 24 h recovered plants, respectively. This approach was used to allow the separation of samples from all three treatments of each genotype in one single gel under identical separation conditions to minimize false positive results due to differences in the running conditions between gels, which occasionally lead to misidentifications of presumably up- or downregulated proteins by automated scanning systems. The criterion for selecting proteins for MALDI-TOF-MS was a 1.5-fold threshold change in relative fluorescence signal intensity [20]. In # 11434, out of the 650 detected spots on the 2D-PAGE, 12 protein spots were upregulated and 6 were downregulated in response to drought stress while 24 hours following recovery 13 protein spots were upregulated and 5 were downregulated. Out of 630 detected spots in # 11431, 19 protein spots were upregulated and 4 were downregulated following drought stress and no protein spots were upregulated and 2 protein spots were downregulated after 24 hours of recovery.  Figure 2 and Supplementary Figure 1 in Supplementary Material (available online at http://dx.doi.org/10.1155/2014/395905) represents the position of differentially expressed protein spots for both accessions in 2D gels that were picked for MALDI-TOF-MS analysis. Proteins were categorized into groups according to their function [23]. The proteins identified with MALDI-TOF-MS; their accession numbers on the NCBI database and their value of alteration in comparison to control values are indicated in Table 1.

### 3.3. Changes in the Group of “Metabolism” Proteins in Response to Drought and Recovery Treatments

The group of metabolism-related proteins [23] included the alteration of methionine synthase (spots 6, 7), S-adenosylmethionine synthase (spot 16), and P-(S)-hydroxymandelonitrile lyase (spot 29). Methionine synthase (spot 6) was upregulated in both # 11434 and # 11431 following drought stress, while the more basic isoform (spot 7) was upregulated only in # 11431. Following recovery, expression levels remained upregulated in #114134 and returned to control values in # 11431. Methionine synthase has been found to increase under drought stress in roots of wild watermelon [24]. Several environmental stresses have been shown to cause changes in the pool of various free amino acids in plant cells from* Arabidopsis* and rice [25, 26]. The activation of methionine synthase is an early response to drought since increased flux through the pathway provides a source of methyl groups for secondary metabolism under stress. Thus, the increase or maintenance of high levels of methionine synthase may reflect more active methionine and osmoregulant metabolism [27]. S-Adenosyl-L-methionine synthase has been found to be upregulated in response to drought in both # 11434 and # 11431, remaining increased in # 11434, and reduced to the level of control 24 hours following recovery in #11431. It catalyzes the syntheses of S-adenosyl-L-methionine (SAM) from L-methionine and ATP. SAM is the major methyl-group donor for many transmethylation reactions in both prokaryotes and eukaryotes [28, 29]. Plants produce several secondary products that include one or more methyl groups added during their biosynthesis by methyltransferases, many of which use SAM as the methyl-group donor. SAM is also a precursor for the biosynthesis of polyamines which are involved in response to abiotic stresses [30]; in particular the polyamine spermine which is synthesized from putrescine via two steps using SAM was shown to have a protective role against drought stress [31]. SAM is further involved in lignin biosynthesis, a major step accelerated with lignification of water stressed* Sorghum* roots and salt stressed maize plants [32, 33]. P-(S)hydroxymandelonitrile lyase was upregulated in # 11431 following drought stress, but not following recovery. Hydroxynitrile lyases are involved in cyanogenesis, release of hydrogen cyanide from damaged tissues, and part of a defense mechanism against herbivores or fungi, respectively [34, 35]. Their upregulation in response to drought stress might reflect the damage of cells of the sensitive genotype or be explained as a cell death mechanism in response to drought stress.

### 3.4. Changes in the Group of Proteins Involved in Energy Metabolism

Two key enzymes of the C4-pathway showed significant changes in their abundance. For both lines drought stress induced an upregulation of PEPC content and a downregulation of NADP-ME. For* Sorghum bicolor*, three different PEPC isoforms are described which are expressed and regulated differentially [36]. The analysed isoform in this work was identified as the C4-Isoform (CP47). PEPC catalyses the primary carbon fixation in the mesophyll cells of C4 plants. Beyel and Brüggemann [37] investigated possible mechanisms of PEPC-regulation under drought stress for* S. bicolor*. First, C4 plants have to maintain a malate gradient to provide a carbon flow from the mesophyll to the cells of the bundle sheath [38]. It is therefore likely that the observed increased overall malate concentrations in* S. bicolor* under drought stress also imply a high malate concentration in the mesophyll cells. PEPC is activated via phosphorylation by a light-induced kinase (PEPC-kinase). Malate is an important inhibitor of the activity of PEPC as well as of PEPC-kinase. At a physiological pH of 7.3 malate concentrations of 5 mM can cause an 80% reduction of the PEPC-activity. In addition, the inhibitory effect of malate could be enhanced by drought-induced pH decrease [37]. The authors concluded that, despite high* in vitro* PEPC activities in drought stressed* S. bicolor*, the* in vivo* activities may be low enough to limit photosynthesis. From the regulation mechanisms of the PEPC-activity different conclusions can be drawn. On the one hand plants may react with a compensation of decreased* in vivo* PEPC-activity by an increasing amount of the enzyme, explaining the increases of the PEPC isoforms of spots 1–4 in Figure 2. On the other hand, these isoforms may only showthe unphosphorylated, inactive population of the PEPC. Phosphorylation of a protein causes a decline of the PI.  Figure 2 shows a much more prominent spot of PEPC-equal mass (100 kDa) in the acid part of the 2D-Gels, and it is likely that this spot represents the active population of the PEPC, while only the unphosphorylated or otherwise modified isoforms spots underwent concentration changes and were analysed. The changes of the abundance of the prominent spot were analysed subsequently and revealed a 1.7-fold decrease for # 11431 under drought stress, while its abundance in the recovered plants of both lines and in the stressed plants of # 11434 did not change strongly. Absence of the positive identification via mass-spec, however, has to be noticed. In future experiments, measurements of the* in vitro* PEPC-activity under identical experimental conditions in the two Egyptian landraces would help to interpret the results from the DIGE analysis. For NADP-ME both lines showed a decrease in abundance under drought stress (spots 9–11 for # 11434 and spot 11 for # 11431). As for the PEPC there are multiple spots identified as NADP-ME which differ in their PI. Beyel [39] and Alfonso and Brüggemann [40] showed a moderate reduction of the NADP-ME activity for* Panicum bulbosum* and* Sorghum bicolor* under drought stress. Downregulation of the NADP-ME activity leads to a decreased decarboxylation rate and a lowered malate consumption. This fact may explain the described accumulation of malate [37]. The lower decarboxylation rate in the bundle sheath cells may also influence total photosynthesis rate (Calvin-Cycle).

Pyruvate phosphate dikinase, PPDK (spot no. 30), was upregulated upon drought treatment only in # 11434 both under drought and following recovery. However, the MALDI-TOF-MS data analysis did not indicate if it is the cytoplasmic or the chloroplastic form of the enzyme. The enzyme has been regarded as a putative rate limiting factor for C4 photosynthesis in* Sorghum* and other NADP-ME-C4 species under control conditions [37, 40, 41]. Under drought stress,* in vitro* activities exceeded the need for the observed photosynthesis rates. In naturally senescing leaves of C3 plants, both the cytosolic and chloroplastic isoforms of PPDK are upregulated: while cytosolic PPDK accumulates preferentially in veins, chloroplastic PPDK also accumulates in mesophyll cells [42]. In* Arabidopsis*, overexpression of PPDK during senescence can significantly accelerate nitrogen remobilization from leaves and thereby increase rosette growth rate and the weight and nitrogen content of seeds [42]. This function might be implemented as an efficient mobilization of metabolites from old to young leaves for plant survival under drought.

Changes in the levels of proteins that have an impact on photosynthesis, glycolysis, and TCA-cycle are also observed. Fructose-1,6-bisphosphate aldolase, FBA, is found in a cytosolic and a plastidic isoform in plants [43]. #11431 revealed changes of both isoforms (upregulation of the cytosolic, downregulation of the plastidic isoform, spots 17, 23, and 24) under drought stress. # 11434 showed a downregulation of the plastidic form only (spots 23, 24). Depending on the cellular localization, FBA has different functions. In the chloroplast the enzyme catalyzes the linkage of dihydroxyacetonephosphate and glycerine-3-phosphate to fructose-1,6-bisphosphate and initiates the regeneration of ribulose-1,5-bisphosphate, the CO_2_-acceptor of the Calvin cycle. The downregulation of plastidic FBA indicates a disturbance of the carbon fixation. Haake et al. [44] showed for transgenic tomatoes that a small decline of the FBA activity leads to lower rates of photosynthesis. Also the downregulation of glycerinaldehyd-3-phosphate dehydrogenase (GAPDH) influences photosynthesis. GAPDH is found in plastidic and cytosolic isoforms (spot no. 19, 20). In chloroplasts GAPDH catalyzes the reduction of 1,3-bisphosphoglycerate to produce glycerinaldehyd-3-phosphate. In conclusion, drought-induced effects on the reductive as well as on the regenerative functions of the Calvin cycle have been observed. The downregulation of FBA and GAPDH under drought stress has recently also been observed by Gong et al. [45] for tomato.

In contrast to the plastidic isoforms, the cytosolic FBA in # 11431 (spot 17) and GAPDH in # 11431 and # 11434 (spot no. 20) were found to be upregulated. In cytoplasm both enzymes play a role in glycolysis. Meanwhile, cytosolic aldehyde dehydrogenase which may also play a role in the detoxification of aldehydes which are produced under drought stress [46].

In addition, the stressed plants of # 11431 indicated an increased amount of aconitase (spot no. 5). This enzyme catalyzes the reaction from citrate to isocitrate in the TCA-Cycle. Besides its function in NADH production via the TCA-cycle, isocitrate is essential for the glyoxylate-cycle and after oxidation to alpha-ketoglutarate it is used in the assimilation of nitrogen [47].

An upregulation of enzymes of glycolysis and respiration has most recently also been reported by Zhao et al. [48] for* Cynodon sp.* under drought stress, while photosynthetic carbohydrate production was suppressed. Plants may be forced to increase catabolic reaction to maintain the metabolic and energetic homeostasis of the cells.

### 3.5. Changes in the Group of Proteins Relevant to Protein Destination and Storage

The chloroplastic form of heat shock protein 60 (Hsp60, spots 12–14) and disulfide isomerase (spot 32) were upregulated following drought stress after 24 hours of recovery in # 11434. Hsp60 is important in the assembly of plastid proteins such as Rubisco [49, 50]. It was suggested to be involved in folding and aggregation of many proteins that are transported to chloroplasts and mitochondria [51]. It binds different types of proteins after their transcription and before folding to prevent their aggregation [52]. In addition, another protein with chaperone functions, protein disulfide isomerase (PDI), was upregulated in drought stressed # 11434. PDI catalyzes disulfide bond formation in the endoplasmic reticulum and assists in protein folding [50] and has been reported to be upregulated in bentgrass in response to drought stress and* Thellungiella rosette* in response to cold stress [27, 53]. Pepsin/retropepsin dependent aspartate protease, APs, (spot 18) was only upregulated in # 11431 following drought treatment. APs have been implicated in protein processing and/or degradation in different plant organs, as well as in plant senescence, stress responses, programmed cell death, and reproduction [54]. The expression of APs in the drought sensitive line might reflect the abundance of degraded proteins under drought stress. Similarly, mitochondrial processing peptidase, MPP, (spot 31) was upregulated only in # 11431 following drought treatment. MPP is responsible for removing the presequence upon import via proteolytic cleavage and additionally it has a metalloprotease feature [55]. Nucleoredoxin, NRX, (spot 8) was upregulated following drought and recovery in # 11434 while it was only upregulated following drought in # 11431. NRX was isolated from maize [56]. This protein is located in the nucleus where it presumably catalyzes redox reactions. The presence of this protein in developing kernels indicates that it could regulate the activity of transcription factors, presumably by altering their redox state. This function might be extended to plants under drought stress.

### 3.6. Changes in the Groups of Transcription and Protein Synthesis-Involved Proteins

RNA binding protein (spot 21) was downregulated in # 11431 following drought treatment and recovered to normal level following recovery, indicating RNA synthesis inhibition in the drought sensitive genotype. On the other hand, 40S ribosomal protein S3 (spot no. 28) was upregulated in # 11343 following recovery. One of its important functions is to maintain the stability of 40S ribosomal proteins. Elongation factor *α*, EF *α*, (spot no. 15) was upregulated in both genotypes after drought stress and remained upregulated during recovery only in # 11434. EF1*α* is responsible for efficient protein synthesis. These results might indicate more efficient RNA transcription and protein synthesis in the drought tolerant genotype especially following recovery.

### 3.7. Proteins with Unclear Classification

Several proteins were upregulated in response to drought stress and following recovery (Table 1). Of these is the ABA stress- and fruit-ripening induced like protein (spot 22). It was found to be upregulated in both the drought sensitive and tolerant genotypes. However it remained upregulated following the recovery of the tolerant genotype. It was suggested that ABA stress- and fruit-ripening induced like protein might adjust the activity of other gene products via its DNA-binding activity or it might function as a protective agent against DNA damage at the earlier stages of stress [57].

## 4. Conclusion 

The drought tolerant genotype proteome analysis indicated that the combined activities of several protein groups may enable the plants to tolerate drought stress and efficiently recover after removing the stress conditions. An efficient mechanism for protein stability, reallocation of metabolites to the newly developed structures, and efficient protein synthesis are the most characteristic features obtained from this study. On the other hand, elements of cell death combined with the production of proteases were the most obvious characteristic for the drought sensitive genotype.

## Supplementary Material

Aliquots of 50 *µ*g of protein samples extractions were labeled with Cy-2, control, Cy-3, drought treatment or Cy-5, recovery treatment, using Cy-Dye DIGE Fluor Minimal Dye Labeling Kit (GE Healthcare) following the manufacturer's recommendation. Following electrophoresis as described in the materials and methods part, gels were scanned using a Typhoon 9410 scanner (GE healthcare). Images were analyzed using DeCyder 2-D Differential Analysis Software (GE healthcare) to identify up and downregulated proteins. Only proteins with 1.5-fold threshold change in relative fluorescence signal intensity are indicated and further subjected for MALDI-TOF-MS analysis.

## Figures and Tables

**Table 1:** MALDI-TOF-MS results of picked spots from control, drought treatment and recovery of drought tolerant, # 11434, and susceptible, # 11431, genotypes and their expression values in comparison to control. The search carried out against the entire NCBInr database; SID: spot identification number; Mr/pI: calculated molecular weight and isoelectric point of predicted proteins; *S*: Score;  *protein scores greater than 84 are significant (*P* < 0.05); *C*%: percentage of coverage; *M*: number of peptides matched; *D*: drought; *R*: recovery.

Spot ID	NCBI Acc. no.	Protein name	Mr/pI	S*	%*C*	*M*	# 11434		# 11431	
							*D*	*R*	*D*	*R*
01: Metabolism										
6	gi/18483235	Methionine synthase/*Sorghum bicolor *	84135/5.93	96	8%	8	6.7	1.6	6.6	
7	gi/18483235	Methionine synthase/*Sorghum bicolor *	84135/5.93	102	12%	12			5.5	
16	gi/226502947	S-adenosylmethionine synthase l *Z. mays *	42986/5.57	193	19%	10	1.7		1.5	
29	gi/666089	P-(S)-hydroxymandelonitrile lyase *Sorghum bicolor *	41796/4.72	154	13%	8			3.6	
02: Energy										
1	gi/242096062	Hypothetical protein Sorbidraft of *S. bicolor* homologues to putative C4 phosphoenolpyruvate carboxylase/*Saccharum officinarum* CAC08829.1	108988/5.89	306	29%	28	2.1		3.3	−2
2	gi/242096062	Hypothetical protein Sorbidraft of *S. bicolor* homologues to putative C4 phosphoenolpyruvate carboxylase/*S. officinarum* CAC08829.1	108988/5.89	504	30%	33			1.7	
3	gi/242096062	Hypothetical protein Sorbidraft of *S. bicolor* homologues to putative C4 phosphoenolpyruvate carboxylase/*S. officinarum* CAC08829.1	108988/5.89	331	29%	30			2.3	−2
4	gi/242096062	Hypothetical protein Sorbidraft of *S. bicolor* homologues to putative C4 phosphoenolpyruvate carboxylase/*S. officinarum* CAC08829.1	108988/5.89	354	26%	30			2.4	
5	gi/242080811	Hypothetical protein Sorbidraft of *S. bicolor* homologues to aconitate hydratase of *Oryza sativa* Japonica Q6YZX6.1	108402/6.41	101	11%	13			1.8	
9	gi/242051769	Hypothetical protein Sorbidraft of* S. bicolor* homologues to chloroplast NADP-dependent malic enzyme *Z. mays* NP_001105313.1	69904/6.23	181	28%	21	−2.8	−2.4		
10	gi/242051769	Hypothetical protein Sorbidraft of* S. bicolor* homologues to chloroplast NADP-dependent malic enzyme *Z. mays* NP_001105313.1	69904/6.23	199	25%	18	−2.8	−2.7		
11	gi/242051769	Hypothetical protein Sorbidraft of* S. bicolor* homologues to chloroplast NADP-dependent malic enzyme *Z. mays* NP_001105313.1	69904/6.23	193	26%	18	−2.8	−2.3	−2	
17	gi/242059597	Hypothetical protein Sorbidraft of* S. bicolor* homologues to fructose-1,6-bisphosphat aldolase, cytosol *Z. mays* NP_001105336.1	38990/6.96	278	23%	12			1.6	
23	gi∣195634659	Plastid fructose-1,6-bisphosphate aldolase/*Z. mays *	41924/7.63	607	37%	22	−2.2	−1.9	−2.4	
24	gi∣195634659	Plastid fructose-1,6-bisphosphate aldolase, *Z. mays *	41924/7.63	395	34%	19	−2.1	−1.9	−1.8	
19	gi∣108705994	Plastid glyceraldehyde-3-phosphate dehydrogenase/*O. sativa* Japonica	34024/4.99	121	19%	11	−1.5			
20	gi/255540341	Cytosolic glyceraldehyde-3-phosphate dehydrogenase/*Ricinus communis *	36930/7.10	157	20%	14	1.6	1.7	1.7	
30	gi/30385668	Pyruvate phosphate dikinase/*Sorghum bicolor *	103021/5.68	94	16%	17	1.9	1.6		
04: Transcription										
21	gi/242085078	Hypothetical protein Sorbidraft of* S. bicolor* homologues to RNA-binding protein/*Arabidopsis thaliana* NP_172405.1	42324/8.89	438	40%	25			−2	
28	gi∣195637410	40S ribosomal protein S3/*Z. mays *	25605/9.4	117	46%	12		1.8		
05: Protein synthesis										
15	gi∣195620072	Elongation factor alpha/*Z. mays *	49534/9.15	225	21%	14	8.5	5	7	
06: Protein destination/storage										
8	gi∣162459902	Nucleoredoxin 1/*Z. mays *	64058/4.8	168	16%	12	4.4	4.9	4.5	
12	gi/242094438	Hypothetical protein Sorbidraft of* S. bicolor* homologues to Heat shock protein 60	61927/5.47	148	31%	16		2.1		
13	gi/242094438	Hypothetical protein Sorbidraft of* S. bicolor* homologues to Heat shock protein 60	61927/5.47	161	29%	14		2.2		
14	gi/242094438	Hypothetical protein Sorbidraft of* S. bicolor* homologues to Heat shock protein 60	61927/5.47	126	19%	12	1.9	2.3		
18	gi/242041951	Hypothetical protein Sorbidraft of* S. bicolor* homologues to pepsin/retropepsin like aspartat protease/*Z. mays* ACG35399.1	42799/8.96	79	14%				2	
31	gi/242056107	Hypothetical protein Sorbidraft of* S. bicolor* homologues to mitocondrial processing peptidase/*Z. mays* NP_001150614.1	54054/6.24	198	28%	12			1.7	
32	gi/145666464	Protein disulfide isomerase/*Z. mays *	56921/5.01	174	19%	18	2.1	2.5		
12: Unclear classification										
22	gi/242075782	Hypothetical protein Sorbidraft of* S. bicolor* homologues to ABA stress- and fruit-ripening inducible-like protein *Z. mays* CAA72998.1	28466/4.92	258	65%	22	3.6	4.5	7	
25	gi/242035869	Osr40Sc1 like protein	39718/6.27	66	9%	3			5.2	
26	gi/242035869	Osr40Sc1 like protein	39718/6.27	201	13%	6	3.6	3.3	3	
27	gi/242056773	Hypothetical protein Sorbidraft/*S. bicolor *	31577/6.06	205	23%	9	3.6	2.9	4.6	
33	gi/242095250	Hypothetical protein Sorbidraft/*S. bicolor *	41333/4.91	222	37%	14	1.9	2.3		
