# Supplementary material for: Comparative Analysis of Sorghum bicolor Proteome in Response to Drought Stress and following Recovery
Source: Int J Proteomics. 2014 Oct 1;2014:395905. doi: 10.1155/2014/395905 (PMC4198819; doi:10.1155/2014/395905)
Supplement: Supplementary file 1 — Aliquots of 50 µg of protein samples extractions were labeled with Cy-2, control, Cy-3, drought treatment or Cy-5, recovery treatment, using Cy-Dye DIGE Fluor Minimal Dye Labeling Kit (GE Healthcare) following the manufacturer's recommendation. Following electrophoresis as described in the materials and methods part, gels were scanned using a Typhoon 9410 scanner (GE healthcare). Images were analyzed using DeCyder 2-D Differential Analysis Software (GE healthcare) to identify up and downregulated proteins. Only proteins with 1.5-fold threshold change in relative fluorescence signal intensity are indicated and further subjected for MALDI-TOF-MS analysis. [file 395905.f1.pdf]

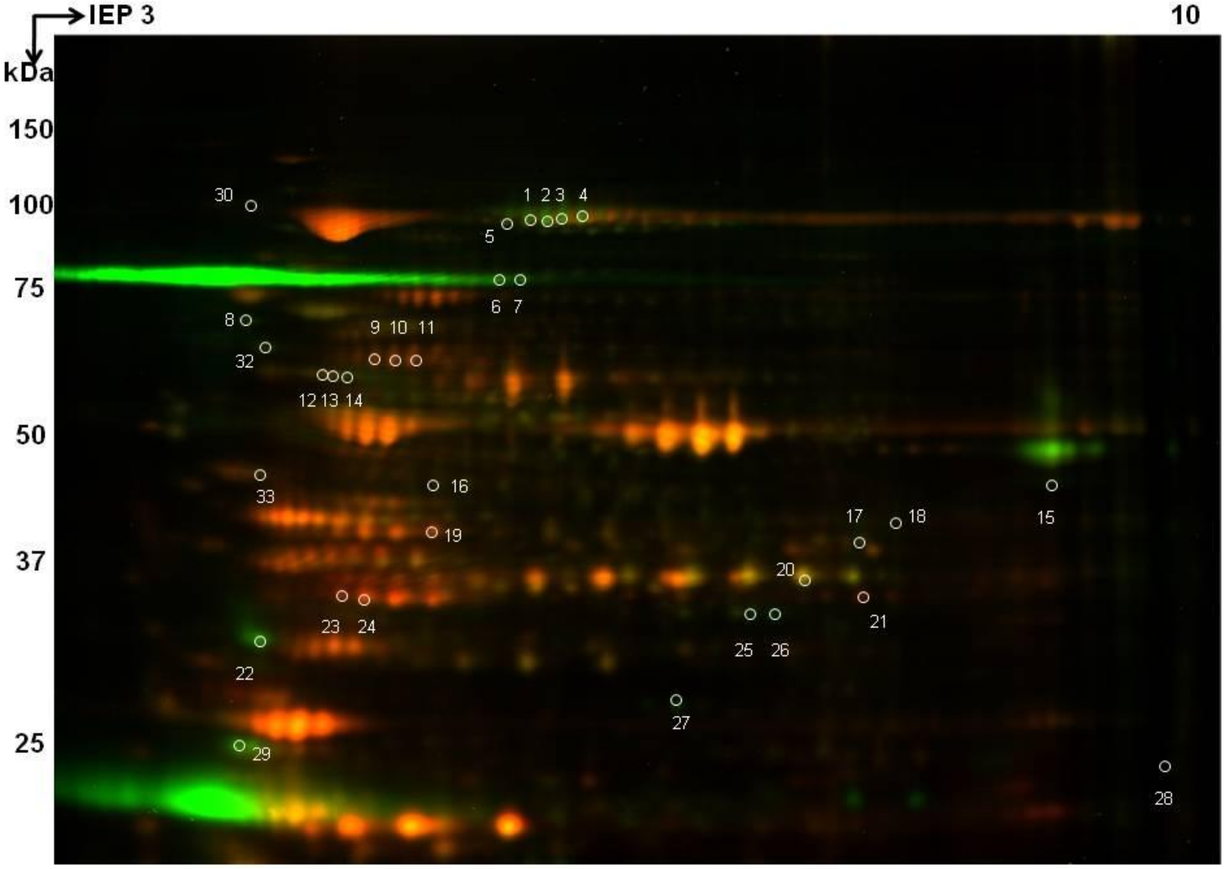

Supplementary Figure 1: Cy-Dye image of differentially expressed Sorghum proteins on 10% SDS-PAGE following separation on 24 cm non-linear strips pH3-10 and scanning. Cycles indicating the positions of up and downregulated proteins spots identified in this study in both genotypes after drought stress and following recovery.
